# Metaplastic breast cancer: histologic characteristics, prognostic factors and systemic treatment strategies

**DOI:** 10.1186/2162-3619-2-31

**Published:** 2013-11-14

**Authors:** Theresa L Schwartz, Harveshp Mogal, Christos Papageorgiou, Jula Veerapong, Eddy C Hsueh

**Affiliations:** 1 Department of Surgery, Saint Louis University School of Medicine, 3635 Vista Avenue—DT 3rd floor, Saint Louis, MO 63110, USA; 2 Department of Internal Medicine, Division of Medical Oncology, Saint Louis University School of Medicine, 3655 Vista Avenue, Saint Louis, MO 63110, USA

**Keywords:** Metaplastic breast cancer, Metaplastic, Basal like breast cancer, Carcinosarcoma

## Abstract

Metaplastic breast cancer (MBC) is a rare subtype of invasive breast cancer that tends to have an aggressive clinical presentation as well as a variety of distinct histologic designations. Few systemic treatment options are available for MBC, as it has consistently shown a suboptimal response to standard chemotherapy regimens. These characteristics result in a worse overall prognosis for patients with MBC compared to those with standard invasive breast cancer. Due to its rarity, data focusing on MBC is limited. This review will discuss the clinical presentation, breast imaging findings, histologic and molecular characteristics of MBC as well as potential future research directions.

## Introduction

Metaplastic breast cancer (MBC) is a rare and histologically diverse subtype of breast carcinoma. It accounts for less than 1% of all breast cancers [1,2]. MBC was not officially recognized as a distinct histologic entity until 2000, and research on this disease process has been limited due to its rarity and the variety of tumor types included in this diagnosis [3]. This category of breast malignancies encompasses tumors in which adenocarcinoma is found to co-exist with an admixture of spindle cell, squamous, chondroid or bone-forming neoplastic cells [4]. Most metaplastic cancers are estrogen receptor (ER), progesterone receptor (PR) and Her2-*neu* negative [5-7] and tend to have a worse prognosis than other triple negative breast cancers [4,8-10] with fewer therapeutic options. In this review, we discuss the histopathology and clinical features of MBC and their relevance to prognosis and systemic treatment options as well as future directions for research.

### Clinical features

The clinical presentation of MBC has several differences from the presentation of other invasive ductal carcinomas (IDC). The median age at time of presentation ranges in the literature from 48 to 59 years [2,4,7]. MBC more commonly presents as a rapidly growing mass, and it has been consistently reported to present larger than typical breast cancers, generally greater than 2 cm [2,11-13]. Fixation to the underlying deep tissues or to the skin has been reported in one study in over 20% of patients [11]. MBC presents with axillary nodal involvement less frequently than standard invasive breast cancer, despite the larger tumor size. The incidence of axillary lymph node metastases spans between 6% and 26%, [4,14-17] which is substantially lower than the expected frequency of axillary nodal involvement in larger invasive breast cancers of greater than 50% [18].

The recommendations for diagnostic imaging are the same as that for other palpable masses. All palpable masses should be investigated with both diagnostic mammography and targeted ultrasound. MBC have been described as having a high density on mammogram with either circumscribed, obscured, irregular and/or spiculated margins. Yang et al. reported a more benign appearance on mammography including a round or oval shape and circumscribed margins. The lesions are often non-calcified [19]. If calcifications are present, the pattern is amorphous, coarse, punctuate or pleomorphic [20,21]. Park et al. described a high rate of architectural distortion associated with MBC [21]. The sonographic appearance of MBC has been previously described as a heterogeneous or hypoechoic solid mass or a mixed cystic and solid mass [19-22]. The complex nature of MBC is consistent with cystic degeneration and necrosis found on pathologic evaluation [22,23]. MBC often demonstrate posterior acoustic enhancement, as opposed to the posterior shadowing commonly seen with IDC [22]. The MRI features described for MBC are an irregular mass with speculated margins, often intermediate to increased T2 signal intensity and isointense or hypointense on T1 weighted imaging [20,24]. Velasco et al. reported an increase in T2 hyperintensity in cancers of 91% of patients with MBC [24]. Although T2 hyperintensity is often associated with benign lesions, it can be secondary to necrosis or mucoid production in malignancies.

The incidence of stage IV disease at presentation for MBC is higher than what is seen with IDC. In one single institution study, 10.3% of patients with MBC had metastatic disease at the time of diagnosis, compared to only 0.9% of patients with IDC [25]. An analysis of the National Cancer Database from 2006 demonstrated that patients with MBC were more likely to present with stage III disease (10.6% vs 8.4%) and stage IV disease (4.6% vs. 3.4%) when compared to patients with other invasive breast cancers [26]. An increased risk of local recurrence (LR) has also been reported in multiple studies. Rayson et al. noted a 53% risk of LR at 2 years, consistent with previous studies documenting LR from 35 – 62% in node negative MBC within 5 years of diagnosis [4,14-17]. This is significantly higher than the expected 17 - 20% 5-year LR for other invasive breast cancers [18]. In one study, age younger than 39, presence of skin invasion and presence of squamous cell carcinoma in lymph node metastases significantly increased the risk for LR and cancer related deaths. The hazard ratios (HR) for tumor recurrence increased to 14.1, 24.8 and 2.2, whereas the HR for tumor related death increased to 34.4, 39.1 and 5.6 respectively based on these factors [9].

The response of MBC to systemic chemotherapy has been consistently poor. Neoadjuvant chemotherapy is minimally effective at reducing tumor burden and preventing progression of disease. Chen et al. reported an 83% progression rate in patients who received neoadjuvant chemotherapy. This group also found that, in their single institution retrospective study, no patient responded to anthracycline, vinorelbine or cyclophosphamide based regimens and a partial response was noted in 3 of 17 patients with a taxane based regimen (17.6%) [27]. These results are similar to the MD Anderson Cancer Center data from 2006 which showed only a 10% complete response rate in patients with MBC undergoing neoadjuvant chemotherapy [28]. Efficacy in metastatic disease is also limited. Cardoso et al. documented a 16.7% response rate to chemotherapy in metastatic MBC, whereas that in metastatic IDC was noted to be between 21 and 75% [29]. Due to the low response rate with chemotherapy, surgical intervention should be the first step in management of patients with operable tumors, regardless of tumor size.

### Pathogenesis

#### Histologic characteristics

The term metaplastic carcinoma was first introduced by Huvos et al. [30]. Histologically, it is a poorly differentiated heterogeneous tumor containing ductal carcinoma cells admixed with areas of spindle, squamous, chondroid, or osseous elements. The wide range of microscopic appearance of MBC has resulted in a variety of classifications and designations. The World Health Organization (WHO) classifies MBC into (1) epithelial type and (2) mixed type [31]. Epithelial-type MBC is, in turn, classified into (1) squamous cell carcinoma, (2) adenocarcinoma with spindle cell differentiation, and (3) adenosquamous carcinoma. Mixed type MBC is classified into (1) carcinoma with chondroid metaplasia, (2) carcinoma with osseous metaplasia, and (3) carcinosarcoma [31].

The spindle cell subtype, which is the most common, demonstrates cells forming poorly cohesive sheets of predominant spindle cell morphology. The spindle cell component often resembles a low-grade sarcoma or reactive process such as granulation tissue, which can be challenging to differentiate [1]. The squamous cell carcinoma subtype demonstrates infiltrating squamous carcinoma with polygonal cells, eosinophilic cytoplasm, and possible keratin pearl formation [1,6]. The carcinosarcoma contains both malignant epithelium and malignant stroma [1,22]. The matrix-producing subtype contains overt carcinoma with a transition to cartilaginous and/or osseous stromal matrix without a spindle component [22,32]. MBC with osteoclastic giant cells subtype shows intraductal or infiltrating carcinoma contiguous or mixed with spindle cell or sarcomatous stroma plus osteoclastic cells [1]. See Table 1 for an outlined description of these classifications according to the WHO.

**Table 1:** World Health Organization histologic classification of metaplastic breast cancer

**Epithelial type metaplastic breast cancer**	**Histologic features**
Squamous cell carcinoma	Infiltrating squamous carcinoma with polygonal cells, eosinophilic cytoplasm and possible keratin pearl formation.
Adenocarcinoma with spindle cell differentiation	Poorly cohesive sheets of predominant spindle cell morphology; resembles a low grade sarcoma.
Adenosquamous carcinoma	Glands display varying degrees of squamous differentiation, characterized by pavement-like architecture, prominent intercellular bridges and, to a lesser extent, keratin formation.
**Mixed-type metaplastic breast cancer**	**Histologic features**
Carcinoma with chondroid metaplasia	Immunoreactive for S-100, patchy for cytokeratin while being negative for smooth muscle antigen.
Carcinoma with osseous metaplasia	Infiltrating carcinoma with osseous differentiation of the mesenchymal component.
Carcinosarcoma	Infiltrating carcinoma with a malignant mesenchymal component.

Adapted from Tavassoli FA, Devilee P: World Health Organization classification of tumors: tumors of the breast and female genital organs. Pathology and genetics of tumors of the digestive system. In *World Health Organization Classification of Tumors.* Lyon, France: IARC Press; 2003:37-41 [33].

Tse et al. classified MBC into 3 groups: epithelial-only carcinoma, biphasic epithelial and sarcomatoid carcinoma, and monophasic spindle-cell carcinoma [6]. Wargotz and Norris classified MBC into five subtypes: matrix-producing carcinoma, squamous cell carcinoma, spindle cell carcinoma, carcinosarcoma, and metaplastic carcinoma with osteoclastic giant cells [14,15,18,33,34].

Alternatively, Oberman classified MBC into spindle-cell carcinoma, invasive ductal carcinoma with extensive squamous metaplasia, and invasive carcinoma with pseudosarcomatous metaplasia. He showed a lack of correlation between the microscopic pattern and the prognosis. This paper concluded that pathologic subclassification has no clinical significance, and that MBC should be considered as one entity [35].

#### Molecular/genetic features

Classically, MBC is biphasic and contains both a carcinomatous component (CC) and a heterogeneous sarcomatous component (HSC). Whether the HSC in a single case are all derived from a common precursor is unknown, and whether this precursor is identical to that of the CC is still controversial. Three major theories have been proposed to explain the co-existence of biphasic components [36]. In the collision theory for a biclonal origin, synchronous growth of the CC and HSC from separate progenitor cells collide to form one tumor. In the combination theory for a monoclonal origin, a common multipotential progenitor cell gives rise to both the CC and the HSC. In the conversion/metaplastic theory for a monoclonal origin, the HSC are derived from the CC through a conversion or metaplastic process.

The presence of transitional areas and epithelial differentiation, such as tight junctions or desmosomes in some HSC supports a metaplastic process [37]. Additionally, the co-expression of S-100 (a myoepithelial marker), vimentin (a mesenchymal marker), and/or cytokeratin (an epithelial marker) in both the CC and the HSC is also evidence for a metaplastic process [38]. These findings suggest that the HSC have an epithelial or myoepithelial origin and undergo subsequent metaplastic changes, but definitive genetic evidence for a monoclonal origin is still very limited [36,39,40].

p53 is a nuclear protein with a tumor suppressor function related to sequence-specific DNA binding and repair of damaged DNA. Wild-type p53 has a short half-life and is not detected by immunohistochemistry (IHC), whereas mutant p53 is stable and detectable by IHC. Non-mutational stabilization of p53 with overexpression has also been demonstrated by IHC. The frequencies of p53 mutations and overexpression are both around 20–40% in conventional breast cancer [41-45]. In MBC, even though there is currently no data on the frequency of p53 mutations, the frequency of p53 overexpression may be as high as 61%, suggesting the involvement of p53 in the pathogenesis of MBC [38]. In addition, p53 alterations might be part of the mechanism underlying the morphological progression of MBC from ductal carcinoma in situ (DCIS) to CC and HSC [46]. In one study, all evaluated biphasic MBC showed concordant and equivalent staining for p53 between the CC and the HSC. The rate of concordance (100%) is higher than that previously reported (64%) for MBC [47]. Further evaluation of the p53 gene mutation status of CC and HSC revealed four mutations, one in each case. For these four cases, identical mutations between the CC and the HSC were found. Additionally, there were also identical mutations between DCIS, CC, and HSC in one case with an available DCIS component. This result provides strong and convincing evidence of the monoclonality not only between CC and each of the HSC, but also among the DCIS, invasive cancer, and HSC of MBC, since it would be extremely unlikely for these morphologically different tumor components to share identical point mutations if they were from different progenitor cells [47].

The fact that identical p53 point mutations have been identified in both stromal and epithelial components in metaplastic carcinomas in not only breast but also urinary bladder and uterus suggest that the combination or conversion theories, which are not mutually exclusive, are the prime modes of histogenesis of neoplasms in these organs [48,49]. There are immunohistochemical and molecular studies that support the hypothesis that the epithelial component is actually the driving force behind the high proliferation rate in these metaplastic carcinomas. For example, vascular endothelial growth factor expression [50] and matrix metalloprotease 7, [51] both of which contribute to invasiveness of tumor, were much more highly expressed in the epithelial aspect of carcinosarcomas.

In one study, transcriptional profiling was performed using half-a-genome oligonucleotide microarrays to elucidate genetic profiles of MBC and their differences to those of IDC using discarded specimens of 4 MBC and 34 IDC. Unsupervised clustering disclosed distinctive expression profiles between MBC and IDC. Supervised analysis identified gene signatures discriminating MBC from IDC as well as between MBC subclasses. Notably, many of the discriminator genes were associated with down regulation of epithelial phenotypes, and with synthesis, remodeling and adhesion of extracellular matrix. Some of them have known or inferred roles related to Epithelial-Mesenchymal transition (EMT). Importantly, several of the discriminator genes were upregulated in a mutant Snail-transfected MCF7 cell known to exhibit features of EMT, thereby indicating a crucial role for EMT in the pathogenesis of MBC. In addition, the identification of SPARC and vimentin as poor prognostic factors have reinforced the role of EMT in cancer progression [52]. The use of certain microRNAs, most notably miR-200f, has also been described. This microRNA is an important modulator of EMT and is found in low levels in MBC. The substantial decrease in miR-200f expression levels was found to be accompanied by an upregulation of EMT transcriptional inducers, further demonstrating the association between EMT and MBC [53]. This finding has been supported by the work of Hayes et al. which showed evidence of Wnt pathway activation, which results in EMT, in nearly all primary metaplastic carcinomas [54].

By transcriptional profiling, MBC is characterized by low expression of GATA3-regulated genes and of genes responsible for cell-cell adhesion with enrichment for markers linked to stem cell function and EMT. In contrast to other breast cancers, most MBC showed a significant similarity to a “tumorigenic” signature defined using CD44^+^/CD24^-^ breast tumor–initiating stem cell–like cells. MBC is enriched in EMT and stem cell–like features, and may arise from an earlier, more chemoresistant breast epithelial precursor than basal-like or luminal cancers [55]. PIK3CA mutations, EMT, and stem cell-like characteristics likely contribute to the poor outcomes of MBC and further highlight the need for novel therapeutic targets [55].

Several authors have demonstrated that MBC consistently harbor a basal/myoepithelial phenotype, therefore suggesting that they may be part of the morphological spectrum of 'basal-like’ breast carcinomas [56-62]. One of the defining features of basal-like breast cancer is epidermal growth factor receptor (EGFR) overexpression [63,64]. Studies have shown that MBC consistently overexpress EGFR but usually lack Her2-*neu* overexpression and amplification [65-67]. Even though MBC are reported to harbor EGFR overexpression in up to 80% of cases, EGFR gene amplification is the underlying genetic mechanism in up to one-third of cases. Given that MBC are poorly responsive to conventional chemotherapy or hormone therapy regimens and that tumors with EGFR amplification are reported to be sensitive to EGFR tyrosine kinase inhibitors, these findings indicate that further studies are warranted to explore EGFR tyrosine kinase inhibitors as potential therapeutic agents for metaplastic breast carcinomas harboring amplification of 7p11 [65,68]. The potential drug targets that could be used for development of more directed therapy for metaplastic breast cancer are listed in Table 2.

**Table 2:** Potential drug targets for directed therapy against metaplastic breast cancer

**Signaling pathways**	**Possible drug targets**
Epithelial-mesenchymal transition pathway	E-cadherin repressor molecules
	Vimentin
MAP kinase signaling pathway	Raf protein kinase
	Mitogen activated protein kinase kinase (MEK)
	Mitogen activated protein kinases (ERK)
PI3K/AKT pathway	PI3 kinase
	Protein kinase 3 (Akt)
	Mammalian target of rapamycin (mTOR)
Epithelial growth factor pathway	Epithelial growth factor receptor (EGFR)

Adapted from Hennessy et al. Biphasic metaplastic sarcomatoid carcinoma of the breast. *Ann Oncol. *2006, 17:605–613 [28].

Sebolt-Leopold. Development of anticancer drugs targeting the MAP kinase pathway. *Oncogene.* 2000, 19:6594-6599 [69].

#### Future directions

Research investigating the development of novel systemic therapeutic regimens is paramount. Studies focusing on finding new molecular markers would allow for the creation of clinical trials, especially in the setting of metastatic disease. Unveiling biological prognostic factors for MBC would also advance our understanding of the progression of this aggressive disease.

The observation that MBCs seem to represent a subset of tumors enriched in EMT and cancer stem-cell (CSC) characteristics, may account for their resistance to therapy and propensity to metastasize [55]. Like tumors that arise from CSCs, MBC display high activation of phosphoinositide 3-kinase (PI3K) pathway components and commonly carry mutations in PI3K or loss of phosphatase and tensin homolog (PTEN) [55]. MBCs also show strong correlation with a CSC-derived genomic profile that is heavily weighted for PI3K activity. Like CSC-derived tumors, most MBCs also display high levels of angiogenesis and commonly express VEGF and HIF-1α [70-72]. In vitro studies have demonstrated that treatment with mTOR inhibitors, such as temsirolimus, results in reduced levels of both hypoxia-inducible factor 1 and vascular endothelial growth factor, which further enhances the effects of inhibition of vasculogenesis by bevacizumab [73-76]. This and other agents targeted against these receptors or pathways could potentially be an area of further research to develop novel therapeutic options for patients with MBC [76].

## Conclusions

MBC is a rare subtype of invasive breast cancer that accounts for less than 1% of all diagnoses. It is characterized by a larger tumor size at presentation, lower rates of axillary nodal involvement, higher rates of both local and distant recurrence, higher rates of ER, PR and Her2 negativity as well as a sub-optimal response to systemic therapies when compared to other invasive breast cancers. Further research studies will be required to develop targeted treatments with the goal of improving clinical outcomes.

## Competing interests

The authors declare that they have no competing interests.

## Authors’ contributions

TS conceived of the review article topic, performed the subject matter literature research and participated in drafting the manuscript. HM performed the subject matter literature research and participated in drafting the manuscript. CP, JV and EH all contributed to the drafting of the manuscript. All authors have read and approved the final manuscript.
